# Case report: Low-dose radiation reverses pembrolizumab resistance in melanoma

**DOI:** 10.3389/fonc.2025.1483117

**Published:** 2025-02-12

**Authors:** Ka Hey Agnes Fong, Isaac Ho, Tsz Him So

**Affiliations:** ^1^ Department of Oncology, Princess Margaret Hospital, Hong Kong, Hong Kong SAR, China; ^2^ Department of Clinical Oncology, Queen Mary Hospital, Hong Kong, Hong Kong SAR, China; ^3^ Li Ka Shing Faculty of Medicine, The University of Hong Kong, Hong Kong, Hong Kong SAR, China

**Keywords:** melanoma, low dose radiation (LDR), immunotherapy, pembrolizumab, radiation

## Abstract

Immunotherapy has been the mainstay of the initial systemic treatment for metastatic melanoma regardless of the tumor’s genetic mutation status (Atkins et al., 2022). It is known to offer long-term overall and treatment-free survival benefits, also with generally tolerable side effect profiles. However, upon disease progression on first- and second-line immunotherapy, further systemic treatment options are limited especially for cases without actionable molecular alterations. With emerging evidence suggesting that radiotherapy can enhance the efficacy of immunotherapy via various mechanisms, together with its potential abscopal effect, the possibility of overcoming immunotherapy resistance with radiotherapy is theoretically sound. We report a case of metastatic melanoma which demonstrated a reversal of immunotherapy resistance after the addition of low-dose radiotherapy to progressive tumor. Complete metabolic remission is achieved with durable response observed.

## 1 Introduction

In patients with metastatic melanoma, genetic sequencing has been available in identifying actionable molecular alterations—most commonly mutation in the BRAF gene at V600 codon. Nonetheless, the combination of immune checkpoint inhibitor with anti-programmed cell death-1 protein (anti-PD1) and anti-cytotoxic T-lymphocyte-associated protein 4 (anti-CTLA4) remains the recommended choice of initial therapy in treatment-naïve, immunotherapy-eligible patients (1, 2), regardless of genetic mutation status (3).

Even though there are remarkable durability of disease control and survival benefits in melanoma patients treated with immunotherapy, there is still a substantial proportion of 40%–50% patients who showed a lack of response to this treatment (4–6). The development of immunotherapy resistance is also inevitable in the initially responding group. Subsequent treatment options are limited especially in patients who are not eligible for the targeted agents. The proposed immunomodulatory effect of low-dose radiotherapy (L-XRT) has shed lights of hope to these patients. The addition of radiotherapy to immunotherapy is increasingly recognized as a potential method to enhance the anti-tumor efficacy and overcome resistance (7, 8). This phenomenon is supported by various pre-clinical models and is also observed in some clinical trials. In addition to the localized effect of radiotherapy, the potential of radiation-induced abscopal effect and the generation of immune memory are also being explored with favorable observations by far. It is therefore plausible to postulate that L-XRT can improve both the local and systemic control of melanoma treated with immunotherapy.

Here we present a case of a patient with metastatic melanoma which demonstrated reversal of immunotherapy resistance, with subsequent exceptional treatment response to the combination of L-XRT and anti-PD1 therapy.

## 2 Case description

In January 2021, a 63-year-old man with a history of metabolic syndrome and deranged renal function presented with a left big toe mass underneath the nail. He was diagnosed with left big toe melanoma subsequently and underwent left big toe resection with left groin lymph node dissection in February 2021. The pathology results confirmed melanoma with lymph node metastases. BRAF mutation was negative.

He was put on adjuvant nivolumab (240 mg every 2 weeks) for 1 month after the operation. After 16 cycles of nivolumab in October 2021, tumor recurrence was noted with increasing cutaneous nodules over the left thigh and the groin region. The 18F-FDG PET/CT scan found no other metastases. The biopsy results of the left groin lymph node proved a BRAF-negative melanoma recurrence. A next-generation sequencing did not show any driver mutation.

He was started on ipilimumab (80 mg) and pembrolizumab (200 mg), given every 3 weeks, since November 2021. After 4 cycles of dual immunotherapy, the results of PET/CT in 1/2022 showed a mixed response with resolution of the known left groin and thigh lesions, but two new lesions were found over the left knee and the left groin, respectively. Resection of the new nodules was performed, and the pathology results confirmed theseto be metastatic melanoma. He was then continued on maintenance pembrolizumab every 3 weeks. Another PET/CT was performed in May 2022 after 5 more cycles, and it showed partial response with one residual left pelvic lesion. Stereotactic body radiation therapy (SBRT) of 40 Gy over five fractions on alternate days was given to the pelvic lymph node in May 2022. He received a total of 11 cycles of pembrolizumab and was put on drug holiday from June 13, 2022 due to tolerance from immune-related grade 2 skin rash and blisters over the bilateral lower limbs and difficult wound healing.

The interim PET/CT in September 2022 showed no recurrence of disease upon drug holiday. In March 2023, new masses over the small bowel and right peritoneum were noted on PET/CT. A small bowel excision was done on April 13, 2023. The pathology results showed recurrence of melanoma with a 6-cm-sized tumor, multiple mesenteric lymph nodes of 2 to 3 cm, and numerous metastatic nodules over the small bowel mesentery. Next-generation sequencing showed no driver mutations.

**Figure 1:**
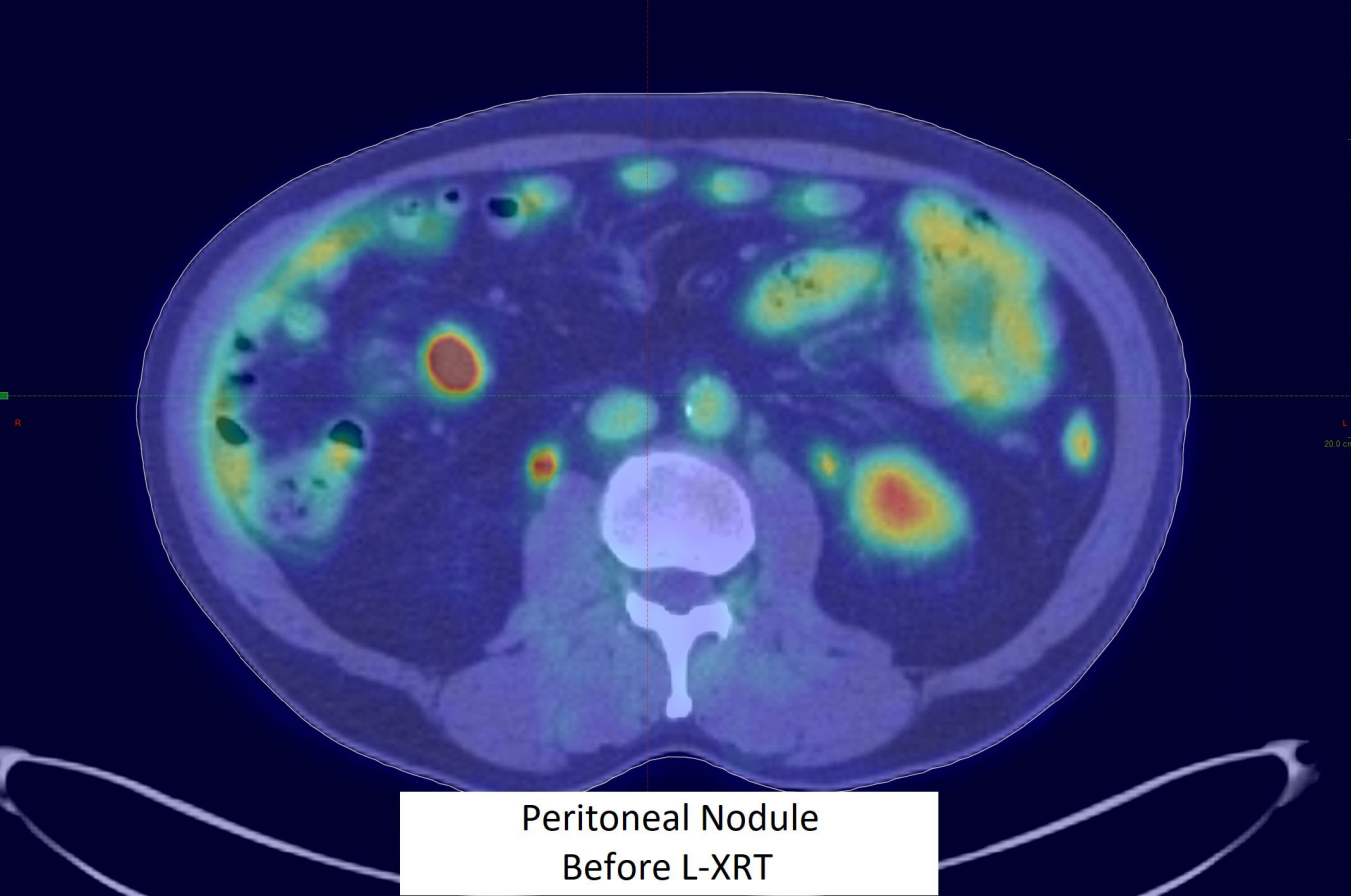
Peritoneal nodule before L-XRT.

He was rechallenged with ipilimumab (75 mg) and pembrolizumab from April 2023, switching to maintenance pembrolizumab alone upon the 5th cycle. The PET/CT results in July 2023 showed a rebound of the disease with a 2.2-cm mesenteric lesion in the peritoneum (**Figure 1**).

**Figure 2:**
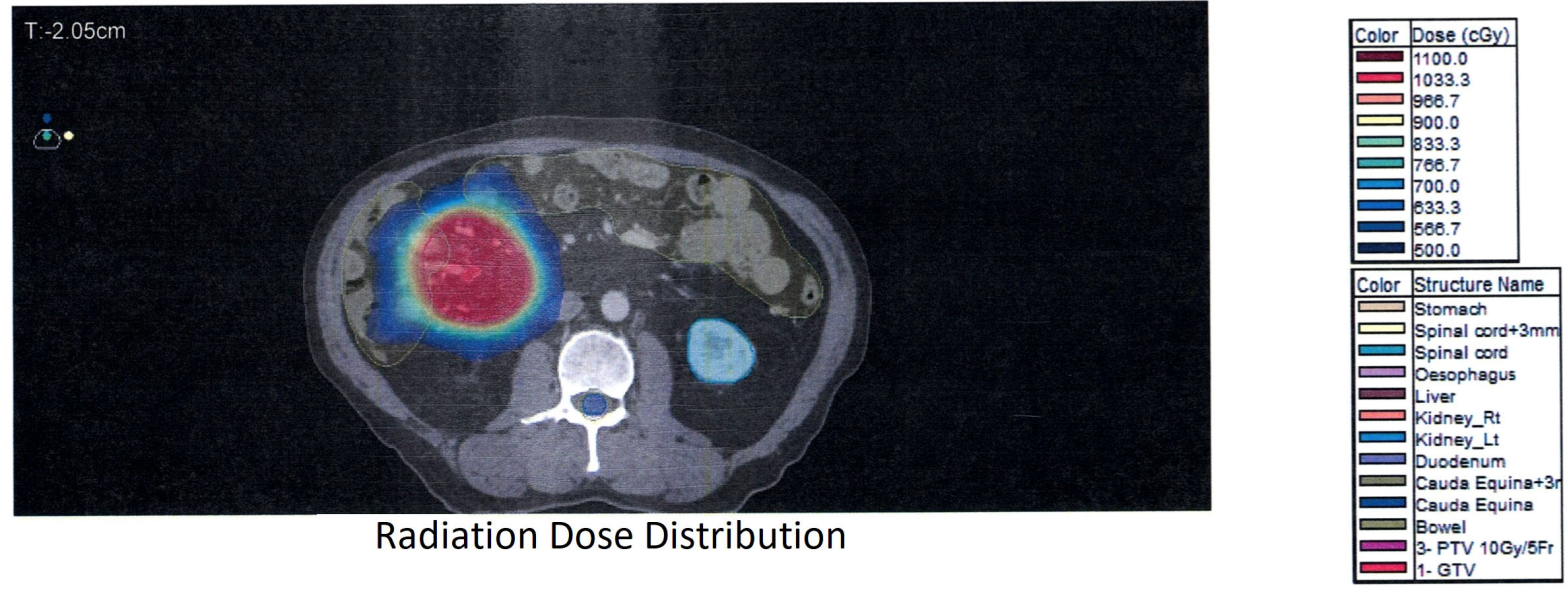
Radiation dose distribution.

A low-dose radiation of 10 Gy over five daily fractions to the mesenteric nodule with 2cm GTV-PTV margin (**Figure 2**) was given from July 21 to 26, 2023. The treatment was well tolerated, complicated only by self-resolving grade 2 diarrhea.

**Figure 3:**
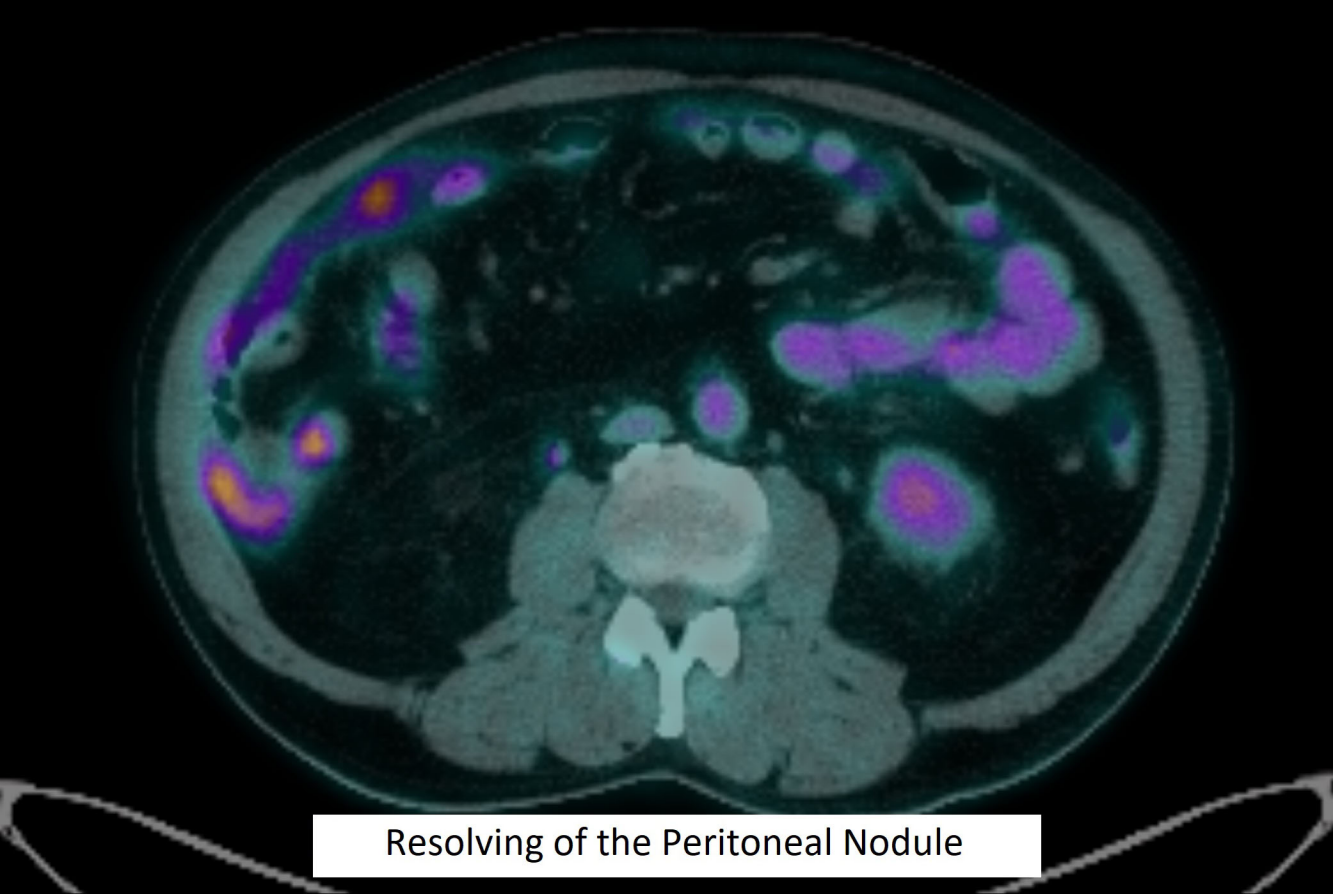
Resolving of the peritoneal nodule.

He continued with maintenance pembrolizumab. The progress CT done 2 months after low-dose RT showed shrinking of the mesenteric nodule from 2.2 to 1.4 cm; no new lesions were identified. The subsequent CT in December 2023 and PET/CT in April 2024 showed complete remission of disease (**Figure 3**).

This patient continued to receive pembrolizumab and finished cycle 19 in June 2024. He tolerated the immunotherapy well and did not have any long-term side effects from the radiation.

## 3 Timeline

**Table 1** below shows a brief description of major events occurred to our patient listed in temporal sequence.

**Table 1:** Highlighted events occurred to our patient in temporal sequence.

Time	Event
**Feb 2021**	Stage III left big toe melanoma, BRAF-negative
**Mar 2021**	Started adjuvant nivolumab, 240 mg every 2 weeks
**Oct 2021**	Left thigh and groin recurrence. biopsy: melanoma BRAF-negative; NGS: no actionable mutation Double immunotherapy ipilimumab + pembrolizumab, 4 cycles
**Jan 2022**	18F-FDG PET/CT: resolution of the left groin and thigh lesions but new left knee skin lesion
**Feb 2022**	Resection of knee lesions: melanoma. NGS: no actionable mutation; pembrolizumab Q3 weeks continued
**May 2022**	PETCT: one residual left pelvic lesion. SBRT 40 Gy/5 Fr given; pembrolizumab continued
**June 2022**	Ceasing of pembrolizumab due to side effects on skin
**Sept 2022**	PET/CT: no active disease found
**Mar 2023**	PET/CT: peritoneum and small bowel recurrence 6 cm mass
**April 2023**	Small bowel resection: melanoma. NGS: no actionable mutation
**April 2023**	Rechallenge double immunotherapy ipilmumab and pembrolizumab for 4 cycles
**July 2023**	New mesenteric 2.2-cm mass and another small peritoneal mass
**July 2023**	Low-dose radiation 10 Gy/5 Fr to peritoneum; pembroliuzmab continued
**Sept 2023**	CT: tumor mass shrunken from 2.2 to 1.4 cm
**Dec 2023**	PET/CT: complete response
**April 2024**	PET/CT: complete response; pembrolizumab continued

Bolded are the month which a highlighted event occurred.

## 4 Diagnostic assessment, therapeutic intervention, and follow-up and outcome

PETCT scan has been used as imaging modality for follow-up diagnostic assessment. Pathological diagnoses were provided on the surgical specimens. Therapeutic intervention included immunotherapy drugs (ipilimumab, pembrolizumab, nivolumab) and radiation (stereotactic body radiation and low-dose radiation). Outcome was complete resolution of the abdominal tumor. No significant side effects or adverse outcomes were found.

## 5 Discussion

This is an intriguing case of metastatic melanoma which demonstrated a promising result of achieving complete tumor resolution with the addition of low-dose radiation therapy (L-XRT) despite its initial resistance to anti-PD1 immunotherapy. This outcome aligns with the emerging evidence which suggests that combining radiotherapy with immunotherapy can enhance antitumor responses and overcome resistance (7, 8).

Radiotherapy has long been recognized for its ability to induce direct cytotoxic effects on tumor cells through DNA damage and subsequent immunogenic cell death (9). However, recent studies have highlighted its potential to modulate the tumor microenvironment (TME) and enhance systemic antitumor immunity. Low-dose radiation, defined as 0.5–2 Gy per fraction for up to 1–10 Gy total, has been shown to overcome the inhibitory stroma within the TME, facilitating better immune cell infiltration and activation (10). This mechanism likely played a crucial role in the observed clinical outcome of our patient.

The RadScopal technique, which involves the strategic use of both high-dose and low-dose radiation, has demonstrated significant potential of RT in enhancing the efficacy of immunotherapy (10). In our patient’s case, the use of L-XRT has likely contributed to the reactivation of the immune response against the tumor. This is supported by findings from various studies where L-XRT was shown to enhance systemic anti-tumor immune responses via multiple mechanisms. Some showed that L-XRT increases the proportion of effector immune cells and reduces inhibitory regulatory T cells in tumors (10–12), while some showed improved T-cell trafficking and infiltration from peritumoral stroma to the intraepithelial tumor compartment after L-XRT (13–17). Notably, it is known that the presence of T-lymphocyte infiltration into melanoma lesions is associated with better prognosis (18). The abovementioned findings are some plausible explanations to the observed reversal of immunotherapy resistance in our patient’s case.

Lattice radiotherapy (LRT) (19) and the Radscopal technique share similarities in their use of spatially modulated radiation to stimulate anti-tumor immune responses while minimizing toxicity to healthy tissues. Both approaches deliver high doses to specific tumor subvolumes, releasing tumor antigens and priming the immune system. However, they differ in application and goals. LRT uses alternating high-dose (peaks) and low-dose (valleys) patterns within bulky tumors, such as large breast cancers (19), to achieve local tumor downsizing and symptom palliation without exceeding organ-at-risk (OAR) tolerance. In contrast, the Radscopal technique combines localized high-dose radiation with systemic immunotherapy, targeting metastatic cancers to enhance systemic immune responses and abscopal effects. While LRT focuses on local control, the Radscopal technique emphasizes systemic tumor control through immune modulation, making them distinct yet complementary strategies in radiation oncology.

Low-dose radiation (L-XRT) plays a crucial role in overcoming the inhibitory tumor stroma, which is a significant barrier to effective immune responses against cancer. The tumor stroma, composed of various cellular and extracellular components, often creates an immunosuppressive environment that hinders the infiltration and function of effector immune cells. L-XRT has been shown to modulate this inhibitory stroma by several mechanisms. Firstly, it reduces the levels of transforming growth factor β (TGF-β), an inhibitory cytokine associated with the protumor M2 phenotype of tumor-associated macrophages (TAMs). By decreasing TGF-β, L-XRT helps to reprogram TAMs from the M2 phenotype to the antitumor M1 phenotype, which supports immune activation and tumor destruction. Additionally, L-XRT enhances the infiltration of effector T cells and natural killer (NK) cells into the tumor microenvironment (TME), thereby promoting a more robust antitumor immune response. This reprogramming of the TME not only facilitates better immune cell trafficking but also creates a cytokine and chemokine gradient that attracts these effector cells to the tumor site. Collectively, these effects of L-XRT help to dismantle the immunosuppressive barriers within the TME, making it more conducive to effective immune-mediated tumor eradication (10, 13).

Apart from the immunomodulatory effect of RT to irradiated tumor, the potential of radiation-induced abscopal effect has been increasingly recognized in recent years as well. The systemic anti-tumor effect is observed to be immune-mediated (20–22). There have also been multiple case reports and trials proving the synergistic phenomenon of RT and immunotherapy in achieving abscopal effect (23–28). The combination of L-XRT with anti-PD1 therapy has been explored in preclinical and clinical settings with encouraging results. This suggests that radiation can potentiate the effects of immune checkpoint inhibitors not only in terms of local control of the irradiated tumor but also leading to regression over distant non-irradiated sites.

Our patient’s complete tumor resolution following the combination of L-XRT and continued anti-PD1 therapy underscores the potential of this approach to overcome immunotherapy resistance. This aligns with the concept that radiation can induce immunogenic cell death, thereby enhancing the visibility of tumor antigens to the immune system and promoting a more robust and sustained antitumor response (29).

Furthermore, the generation of long-term immune memory, as observed in preclinical models with similar combinatorial approaches, suggests that this strategy does not only address the existing tumors but may also provide protection against future recurrences (30). This is particularly relevant for melanoma, a cancer known for its high mutational burden and potential for immune evasion. Nevertheless, the innovative approach of combining immunotherapy with radiation requires careful consideration. This area of research presents certain challenges, as this therapeutic strategy may lead to uncommon adverse effects—for example, cases of bone erosion (31) and neuritis symptoms (32) have been reported.

There are several limitations to our presented case. Firstly, we utilized the higher end dose (10 Gy in 5 Fr) of L-XRT to treat the mesenteric tumor. It remains uncertain whether a lower dose of L-XRT could achieve the same therapeutic effect while reducing the side effect of diarrhea. Secondly, we did not conduct serum tests for immune markers such as TGF-β. Thirdly, a biopsy of the recurrent mesenteric tumor was not performed due to its location, which would necessitate laparoscopy. The patient declined another laparoscopy following a recent bowel resection surgery. Obtaining a tumor sample would have been valuable for studying the tumor microenvironment. These limitations can be addressed in a future prospective study.

In conclusion, the successful treatment of our melanoma patient with peritoneal metastases using L-XRT in conjunction with anti-PD1 therapy highlights the potential of integrating radiotherapy with immunotherapy to overcome resistance and achieve durable responses. This case adds to the growing body of evidence supporting the synergistic effects of these modalities and underscores the need for further research to optimize combination strategies for cancer treatment.

## 6 Patient’s perspective

From my own perspective, undergoing this innovative treatment has truly been a life-changing experience. At first, I was a bit concerned about the idea of receiving low-dose radiation, but the process turned out to be manageable. I experienced mild diarrhea during the treatment, but these were small inconveniences compared to the benefits I gained. Now, I am thrilled to be living a normal, active life—I can go about my daily routines and enjoy time with my family without constantly worrying about my cancer. It is incredible to me that such a simple and relatively low-cost treatment has made such a profound difference in reversing the resistance to my previous immunotherapy. I am genuinely amazed by the results and hopeful that this approach can help others facing similar health challenges. This journey has renewed my enthusiasm for exploring new treatment options and has given me a fresh perspective on the possibilities in cancer care.

## Data Availability

The raw data supporting the conclusions of this article will be made available by the authors, without undue reservation.
